# Cannabinoids and Dementia: A Review of Clinical and Preclinical Data

**DOI:** 10.3390/ph3082689

**Published:** 2010-08-17

**Authors:** Sebastian Walther, Michael Halpern

**Affiliations:** University Hospital of Psychiatry, Bolligenstrasse 111, 3000 Bern 60, Switzerland; E-Mail: Michael.Halpern@gef.be.ch (M.H.)

**Keywords:** cannabinoids, Alzheimer’s disease, Huntington’s disease, Parkinson’s disease, vascular dementia

## Abstract

The endocannabinoid system has been shown to be associated with neurodegenerative diseases and dementia. We review the preclinical and clinical data on cannabinoids and four neurodegenerative diseases: Alzheimer’s disease (AD), Huntington’s disease (HD), Parkinson’s disease (PD) and vascular dementia (VD). Numerous studies have demonstrated an involvement of the cannabinoid system in neurotransmission, neuropathology and neurobiology of dementias. In addition, several candidate compounds have demonstrated efficacy *in vitro*. However, some of the substances produced inconclusive results *in vivo*. Therefore, only few trials have aimed to replicate the effects seen in animal studies in patients. Indeed, the literature on cannabinoid administration in patients is scarce. While preclinical findings suggest causal treatment strategies involving cannabinoids, clinical trials have only assessed the suitability of cannabinoid receptor agonists, antagonists and cannabidiol for the symptomatic treatment of dementia. Further research is needed, including *in vivo* models of dementia and human studies.

## 1. Introduction

Neurodegenerative diseases and dementia have a great impact in today’s aging society, including high costs and burden of disease. Today, about 24 million people suffer from dementia worldwide and the number is expected to double every 20 years [1]. The prevalence rates vary among the different types of dementia. Alzheimer’s disease (AD) is the most common dementia, accounting for 50–60% of all cases. Prevalence rates increase with age [2]. In Parkinson’s disease (PD) the risk for developing dementia is increased 6-fold [3]. Approximately 30% of stroke survivors develop post stroke dementia [4]. Far lower prevalence rates are documented for Huntington’s disease, which is frequently associated with dementia [5]. Although researchers focus on causal treatments, at this moment only symptomatic treatments are available for any type of dementia [4,6,7,8]. 

For more than 4,000 years, the hemp plant has been used in China and India for its medicinal effects. These were recognized in Europe in the 19^th^ century [9]. Research increased tremendously after 1964, when Gaoni and Mechoulam [10] identified the correct chemical structure of Δ^9^-tetrahydrocannabinol (Δ^9^-THC), the main psychoactive compound of marijuana. Later, in the 1990s receptors for cannabinoids were found [11,12]. It would be out of the scope of this article to review the pharmacology of cannabinoids (CB) in general. We recommend existing excellent reviews on the topic [9,13,14,15,16,17,18,19]. In short, endogenous cannabinoids serve as neuromodulators via retrograde signaling [19], they are synthesized on demand from membrane phospholipids [18,20]. Inactivation of endocannabinoids is accomplished either through transport back into the cell or hydrolysis by the enzyme fatty acid amide hydrolase (FAAH) [9,18]. Currently, two cannabinoid receptors are known in the brain, CB_1_ [11] and CB_2_ [12], while there is ongoing discussion as to whether there are even more cannabinoid receptors [9]. Highest densities of CB_1_ were found in the basal ganglia, amygdala, hippocampus and cerebellum [21,22,23,24]. Both CB receptors mediate action via G-protein coupling. Moreover, cannabinoids may activate multifunctional mitogen-activated protein kinases (MAP-kinases) and may regulate phosphatase activity [9]. The mechanism of action for cannabidiol (CBD) is not known. In fact, the phytocannabinoid CBD has only very low affinity to either CB receptor and may elicit anti-inflammatory action as it mimics an inverse CB_2_ agonist [17]. Cannabinoids mentioned in this paper and their classification are given in Table 1. Note, that this table is far from being a complete list of cannabinoids. 

Because of their broad impact on neurotransmission through retrograde signaling and involvement in inflammation, endocannabinoids have been suggested as modulators of various neurodegenerative diseases [9,25,26,27,28,29,30]. However, the growing preclinical data have not yet been influencing the treatment regimes of our patients. Instead, the few clinical trials of dementia with cannabinoid compounds were initiated because the use of marijuana in several neurological and psychiatric disorders has been known for centuries [9]. 

Here, we review the evidence for cannabinoids in common forms of dementia associated with neurodegeneration: Alzheimer’s disease (AD), vascular dementia (VD), Huntington’s disease (HD), and Parkinson’s disease (PD). For better reading, we sorted the results according to the type of research (preclinical *vs.* clinical).

**Table 1:** Cannabinoids mentioned in this paper.

	Name	Mechanism of action
Phytocannabinoids	Δ^9^-Tetrahydrocannabinol (Δ^9^-THC/dronabinol))	CB_1_ and CB_2_ agonist
	Δ^8^-Tetrahydrocannabinol (Δ^8^-THC)	CB_1_ and CB_2_ agonist
	Cannabidiol (CBD)	no activity at CB_1_ and CB_2_, inhibition of AEA uptake and metabolism
Endogenous cannabinoids	Anandamide (AEA)	CB_1_ >> CB_2_ agonist
	2-Arachidonoyl glycerol (2-AG)	CB_1_ and CB_2_ agonist
Synthetic cannabinoids	HU-210	CB_1_ and CB_2_ agonist
	Nabilone	CB_1_ and CB_2_ agonist
	WIN55,212-2	CB_1_ and CB_2_ agonist
	CP55,940	CB_1_ and CB_2_ agonist
	JWH015	CB_2_ selective agonist
	HU-308	CB_2_ selective agonist
	SR141716A	CB_1_ selective antagonist
	AM404	anandamide transport inhibitor
	UMC707	anandamide transport inhibitor
	Arvanil	CB_1_ agonist, vanilloid receptor agonist

## 2. Methods

We performed a PUBMED search in February 2010 using the terms DEMENTIA and CANNABINOID that led to 80 documents. Of those, 27 were reviews, 50 research articles and three case reports. Furthermore, we used the information from the reviews to find additional related papers and performed individual searches for associations between the cannabinoid system and single symptoms of dementia.

## 3. Results and Discussion

### 3.1. Preclinical findings

#### 3.1.1. Alzheimer’s disease

Alzheimer’s disease is characterized by extracellular neuritic plaques of β-amyloid (Aβ) deposits and by intracellular tangles that are formed by hyperphosphorylated tau protein [2,31]. Finally, it is believed that the combination of oxidative stress and abnormal mitotic signaling leads to the neuropathological AD phenotype [32].

A body of literature reports on the involvement of the endocannabinoid system in Alzheimer’s disease pathology [26,27,33]. CB_1_ receptors were found in rat brains in the hippocampus, striatum, cingulate gyrus and entorhinal cortex [34,35]. Especially in the limbic system CB_1_ receptors show high densities, where agonists inhibit γ-amino butyric acid (GABA) release and modulate glutamate release [23,24,36]. Thus, CB_1_ receptors regulate neurotransmitters involved in excitotoxic neurodegenerative processes.

In fact, neurodegeneration in AD includes excitotoxic neuronal death as a result of Aβ-induced neuroinflammation. Activated microglia produce nitric oxide, which in turn inhibits neuronal respiration and thereby leads to glutamate release. As a result, neurons are killed by excitotoxicity [37]. Furthermore, microglia activation and migration seems to be regulated by CB_2_ receptors [38]. However, some of the action is not mediated by CB receptors but is elicited by antioxidant compounds such as cannabidiol (CBD).

##### 3.1.1.1. Effects mediated via cb_1_ and cb_2_ Receptors

In AD brains cannabinoid receptor binding was reduced in the hippocampal formation and caudate [39], whereas the mRNA levels did not differ from controls. Concerning the CB_1_ receptor, one study reported no difference in CB_1_ density around the neuritic plaques [40], while another study found CB_1_ receptor positive neurons to be reduced in areas of microglial activation [41]. The difference may stem from the different brain regions investigated [41]. 

In the hippocampus of rats CB_1_ agonists inhibit the presynaptic release of glutamate via G-protein mechanisms [42], which was later shown to prevent excitotoxicity *in vitro* [43]. In fact, protection against excitotoxicity by the endocannabinoid system was shown be activated on demand [44].

*In vivo N-*methyl-*D*-aspartate (NMDA) injection into the rat cortex leads to a pronounced increase of the endogenous cannabinoid anandamide, which may represent a protective mechanism to restrict neurotoxicity [45]. In line with that finding, *in vivo* models of excitotoxicity demonstrated that the administration of either Δ^9^-THC or anandamide reduced neuronal damage via CB_1_ receptor mediated effects [46,47]. CB_1_ agonists were shown to prevent Aβ-induced neurotoxicity *in vitro* [48]. One mechanism of action is the reduction of nitric oxide production, which in turn led to reduced tau protein hyperphosphorylation [49]. Another mechanism suggested is that the brain-derived neurotrophic factor (BDNF) mediates the neuroprotective effects of CB_1_ agonists [50]. Furthermore, both CB receptor types regulate the release of the interleukin 1 receptor antagonist (IL–1ra) from glia cells, which is in turn essential for the CB mediated neuroprotection [51].

CB_2_ receptors are highly expressed in microglia. In post-mortem AD brains, CB_2_ receptor mRNA was demonstrated to be upregulated in the hippocampus [52] as well as in microglia and astrocytes surrounding neuritic plaques [40]. Indeed, CB_2_ receptors were also expressed within neuritic plaques of AD brains [41]. Therefore, an association of CB_2_ receptors in neuroinflammation was suggested. In fact, CB_2_ receptors in microglia were upregulated by proinflammatory cytokines such as γ−interferon (γ-IFN) and granulocyte macrophage-colony stimulating factor (GM-CSF) in animal models [53,54]. Experimental brain inflammation increased mRNA expression of CB_2_ receptors 100-fold [54]. 

Three potential interventions were identified in experiments targeting CB_2_ receptors. First, CB_2_ agonists suppress the neuroinflammatory process via both, reduction of CD40 expression and reduction of nitric oxide and tumor necrosis factor α (TNF-α) production in activated microglia [53]. Second, *in vitro* models of AD suggested that CB_2_ agonists may lead to β-amyloid removal via stimulation of human macrophages [55] and the suppression of CD40-mediated inhibition of microglial phagocytosis [53]. Third, microglia activation may be reduced by the CB_1_/CB_2_ agonists WIN55212-2 [35] and HU-210 [41]. Furthermore, along with the prevention of microglial activation, CB_1_/CB_2_ agonists led to improved memory performance in rat models of AD and normal aging [34,41]. 

Taken together, CB_1_ agonists may interrupt the mechanisms of excitotoxicity as they reduce glutamate release, and CB_2_ agonists may suppress neuroinflammation and lead to plaque removal. Moreover, one study demonstrated that Δ^9^-THC inhibits the acetycholine esterase *in vitro* and prevents acetylcholine esterase induced Aβ-aggregation [56].

##### 3.1.1.2. Effects of Cannabidiol

Antioxidant effects have been ascribed to CBD [27,33]. Still, the mechanism of action of CBD remains unclear. No specific receptor has been identified and it is hypothesized that CBD influences the metabolism of endocannabinoids such as anandamide [33]. 

CBD was shown to protect against Aβ-induced neurotoxicity *in vitro*. CBD as an antioxidant and anti-apoptotic compound reduced DNA fragmentation, lipid peroxidation, the production of reactive oxygen species, the levels of key enzymes for apoptosis as well as the intracellular calcium [57]. Further, after Aβ-challenge *in vitro* CBD inhibited intracellular signaling pathways and thereby suppressed tau protein hyperphosphorylation [58] and the production of nitric oxide [59]. These results were further corroborated by an *in vivo* model, in which Aβ (1–42) protein was injected in the right dorsal hippocampus of mice. In this experiment CBD dose dependently suppressed the production of proinflammatory molecules, including Interleukin 1β and nitric oxide [60].

In summary, CBD as a nonpsychoactive cannabinoid targets the oxidative stress in AD as well as tau phosphorylation. More animal studies are required to substantiate these findings *in vivo* and to prepare prospective human studies.

#### 3.1.2. Vascular dementia

Vascular dementia develops as a consequence of brain ischemia. In animal *in vivo* models of focal or global cerebral ischemia, several CB_1_ agonists reduced infarct volume and neuronal cell death [61,62,63,64,65,66,67], most likely because of hypothermia and NMDA antagonism [68]. However, some groups reported contradictory findings. CB1 antagonists reduced neuronal death and endogenous cannabinoids increased neuronal damage [69]. Because cannabinoids mediate action mainly via retrograde signaling, it was suggested that in ischemia, CB_1_ activation leads to inhibition of GABA and glutamate release the former resulting in neurotoxic effects and the latter in neuroprotection [68]. Because of the inconsistent findings, no cannabinoid based intervention in cerebral ischemia is at sight. Still, after further research the cannabinoid system may become a target for interventions, as CB_2_ activation may influence stroke outcome [29]. Currently, no data are available on the molecular mechanisms of VD. However, cerebral infarction is the major cause for VD [70].

#### 3.1.3. Huntington’s disease

Huntington’s disease is an autosomal dominant inheritable disorder that leads to excessive body movements and cognitive decline [8]. Worldwide a prevalence of 5–8/100,000 is observed, with highest frequencies in Europe and India. HD patients have longer CAG repeats in the DNA of the huntingtin gene. The neurodegenerative process is driven by neurotransmitter changes (mainly loss of GABA transmission) and focuses on basal ganglia projections [5].

Neuropathological studies have linked the CB receptor density in basal ganglia to the stages of HD. In fact, CB receptors were found to be located within the substantia nigra [71]. In HD brains, CB receptor binding in basal ganglia decreases with disease progression [71,72]. The loss of CB receptors mainly affects striatal projections [73]. During the course of the disease striatopallidal neurons are affected: first projections to the lateral globus pallidus, secondly those to the substantia nigra and finally, the neurons projecting to the medial segment of the globus pallidus. The main neurotransmitters involved are GABA, enkephaline and substance P [72]. An upregulation of GABA receptors in the globus pallidus was found in HD brains and thought to exert a compensatory mechanism to the reduced GABAergic transmission following striatopallidal neurodegeneration [74]. In addition, in the striatum of an HD transgenic mouse model postsynaptic activity was increased. Interestingly, the CB_1_ and CB_2_ receptor agonist HU210 failed to reduce GABA transmission in the striatum of HD mice and even increased postsynaptic activity [75].

Rodent models of HD neurodegeneration have repeatedly demonstrated the link to the cannabinoid system. Transgenic HD mice expressed less CB_1_ receptors in the lateral striatum, within a subset of neurons in the cortex and in the hippocampus compared to age-matched controls [76]. Furthermore, the relative expression level of mutant huntingtin or the length of the CAG repeat or both were found to affect the onset and rate of the decrease of CB_1_ receptor transcription [77]. Likewise, in another transgenic HD mouse model CB_1_ receptor expression in the caudate-putamen and its projection areas were decreased as well as the efficacy of CB_1_ receptor activation in the globus pallidus compared to age-matched controls [78]. Interestingly, transgenic HD mice housed in enriched laboratory environments showed less depletion of CB_1_ receptors in basal ganglia than their counterparts in standard laboratory environments [79].

Alterations of CB_1_ receptor expression may develop in different directions according to the brain region involved. In fact, in a toxic rat model of HD endocannabinoids levels were decreased in the striatum and increased in the ventral mesencephalon, where the substantia nigra is located; both sites of alterations were suggested to contribute to the hyperkinesia seen in HD patients [80].

*In vitro* cell-based assays revealed the potential use of cannabinoids (Δ^8^-THC, Δ^9^-THC, CBD) and caspase inhibitors, because they were able to protect neurons from death caused by an expanded polyglutamine form of huntingtin exon 1 [81]. In contrast, in a toxic rat model of HD the CB_1_ agonist Δ^9^-THC as well as the CB_1_ antagonist SR141716A increased the toxic lesions. The authors suggested that protective and toxic effects may overlap in a dose dependent manner [82]. In fact, the mechanisms are not clear yet. CB_1_ upregulation in HD brains concurred with the upregulation of BDNF in corticostriatal neurons [83]. Furthermore, neuroinflammation seems to be involved in HD as well. The CB_2_ receptor expression increased in the striatal microglia of HD transgenic mice and of HD patients, and CB_2_ agonists reduced neuroinflammation, striatal neuronal loss and motor symptoms in a toxic mouse model of HD [84]. Microglial activation was demonstrated in post-mortem HD brains [85], *in vivo* in HD patients [86] and asymptomatic Huntington gene carriers [87].

In addition, a number of *in vivo* models of HD investigated substances that may reduce hyperactivity [88,89,90]. Indeed, AM404, UCM707 and Arvanil modulate endocannabinoid signalling. AM404 and UCM707 are inhibitors of endocannabinoid uptake, while Arvanil is an inhibitor of the endocannabinoid transporter and a direct CB_1_ agonist. In addition, AM404 and Arvanil are agonists at the vanilloid receptor TPRV1.

In normal and HD human brain CB_1_ positive proliferating cells were detected in the subependymal layer, raising the intriguing possibility that these cells could provide a suitable source of cells for endogenous replacement of lost cells in HD, if they could be mobilized [91]. In summary, CB receptors in the basal ganglia are lost during the disease progression and CB agonism reduced hyperactivity *in vivo*. The role of CBs in HD neuroinflammation remains still unclear.

#### 3.1.4. Parkinson’s disease

PD has a lifetime prevalence of 1.5% and is characterized by progressive motor, cognitive and behavioural disturbances [7]. Preclinical research in PD has focused mainly on neuroprotection, neurotransmission and the neurobiology of dyskinesia. Neuroprotection in PD is mostly mediated via antioxidant properties of cannabinoids. Indeed, the CB_1_/CB_2_ receptor agonist CP55, 940 protected against paraquat toxicity, which induces acute parkinsonism [92]. The mechanism of action however, was not receptor mediated. Instead, the neuroprotection was achieved through inactivation of the oxidative stress responsive Jun-*N*-terminal kainase signalling. As a result, Drosophila melanogaster, which have no cannabinoid receptors, were able to climb again after CP55,940 administration. 

Cannabinoids reduced neuronal damage via various pathways (CB_1_, CB_2_ and CBD) in animal models of neurodegeneration in PD. Δ^9^-THC, a CB_1_ agonist with antioxidant properties, CBD and AM404, an inhibitor of endocannabinoid inactivation with antioxidant properties, ameliorated the effect of nigrostriatal lesions in a PD rat model probably as a result of their antioxidant properties [93]. Likewise, the CB_2_ receptor agonist HU-308 produced a small recovery of nigrostriatal lesions, indicating that the activation of CB2 receptors might also contribute to neuroprotection [94]. However, in a different PD rat model [95] the non-selective CB receptor agonist WIN55, 212-2 ameliorated the effect of nigrostriatal lesions independently of CB_1_ receptor activation, which is in contrast to the former study [94], where it didn’t have any effect. In the same rat model, WIN55, 212-2 and the CB_2_ receptor agonist JWH015 reduced the lesion–induced and potentially deleterious microglial activation [95].

Cannabinoids play an important role in neurotransmission in PD. In a rat model of parkinsonism the dopamine D2 receptor agonist quinpirole caused an alleviation of akinesia, which was reduced by coinjection with the CB receptor agonist WIN 55, 212-2 [96]. In addition, in that same rat model 2AG levels were increased sevenfold in the globus pallidus [97], whereas CB_1_ receptor mRNA expression in the striatum are reduced [98]. Furthermore, in another PD rat model the metabolism of endocannabinoids was impaired with increased striatal anandamide levels and elevated striatal glutamatergic transmission. The elevated glutamatergic transmission was reversed by administration of anandamide membrane transporter (AMT) inhibitors, fatty acid amide hydrolase (FAAH) inhibitors or a CB_1_ agonist [99]. In fact, CB1 agonists were able to decrease glutamate release from afferent terminals in the striatum in post–mortem rat brains [100]. 

CB_1_ antagonists may help to alleviate motor dysfunction in PD. Animal models of PD demonstrated a beneficial effect of CB_1_ antagonists augmenting levodopa in rats [101] and rhesus monkeys [102]. In a PD rat model locomotion was restored by coadministration of the dopamine D2 agonist quinpirole and the selective CB_1_ receptor antagonist SR141716A, which augmented the quinpirole effect [97]. Likewise, in another rat model, the systemic administration of SR141716A exerted an antiparkinsonian effect, but only in rats with very severe nigral lesion (>95%) [103]. However, in a PD primate model, SR141716A failed to alleviate motor deficits, probably due to interspecies differences [104]. Further, in a mild PD marmoset model Δ^9^-THC improved motor deficits. It was therefore suggested that CB_1_ agonists could be the compound of choice in the early symptomatic phase of PD, as CB antagonists would work in a later phase [105].

In animal models of levodopa–induced dyskinesia, coadministration of CB agonists (HU-210 and nabilone) with levodopa reduced dyskinesia [106,107]. Indeed, levodopa reduces extracellular glutamate, an effect that is prevented by CB agonists. Extracellular glutamate is inversely correlated with dyskinesia, *i.e*., higher glutamate levels were seen in animals with less dyskinesia [108].

In summary, cannabinoids may reduce neurotoxicity in PD and CB agonists were shown to reduce dyskinesia. However, results were inconclusive to whether CB agonists or antagonists could alleviate motor symptoms in PD.

### 3.2. Clinical findings

In Alzheimer’s disease, clinically used strategies involve acetylcholine esterase inhibitors and memantine to slow symptom progression. Experimental approaches currently study the use of secretase modulators, Aβ-immunotherapy, Aβ-fibrillisation inhibitors, anti–inflammatory drugs, antioxidants and cholesterol-lowering drugs [2]. Today, there is no causal treatment for HD, PD or VD either [5,7,8,109].

To our knowledge, there are currently no data available on curative treatment of any dementia using cannabinoids [110]. However, a small but growing body of literature reports on the use of cannabinoids in the symptomatic treatment of dementia and neurodegenerative diseases. Interestingly, none of the studies focused on cognition or memory. Instead, behavioral and motor symptoms were approached.

#### 3.2.1. Alzheimer’s disease

Two clinical trials and one case report are available on the topic. The two studies used dronabinol and one case report used nabilone, both substances are CB_1_ and CB_2_ agonists [9]. Volicer and colleagues [111] investigated 15 institutionalized patients with severe dementia who presented with food refusal in a randomized double blind placebo controlled crossover trial of dronabinol 2.5 mg b.i.d. Each period lasted for six weeks. Of the 15 participants three experienced severe side effects (seizures, intercurrent infections) and had to be excluded. Body weight increased and agitation decreased during dronabinol periods. In addition, the authors observed a considerable carry over effect on agitation in those who received active treatment first.

Walther and colleagues [112] used actigraphy and the Neuropsychiatric Inventory (NPI) [113] to investigate the effects of oral dronabinol 2.5 mg administered at 7 PM on night-time agitation and behavioral disturbances in an open label pilot study including six patients with dementia (5 AD and 1 VD). Over two weeks of treatment objectively measured nocturnal motor activity and the NPI total score were reduced, as were the NPI items agitation, aberrant motor behavior, appetite disturbances, irritability and night-time behaviors. This study found no adverse effects during the two week trial period. 

Subsequently, Walther and colleagues started a randomized, double-blind, placebo-controlled, crossover trial of dronabinol 2.5 mg to further evaluate the effects on circadian rhythm and behavioral disturbances in Alzheimer’s disease. The study, however, was aborted due to recruitment failure. Still, two patients were included and both displayed reduced nocturnal motor activity and stabilized circadian rhythms without any side effects during the dronabinol period (Walther *et al*. unpublished data).

Nabilone was used in a patient with Alzheimer’s disease [114] who had been subsequently treated with donezepil, memantine, trazodone, quetiapine, and olanzapine without any impact on the behavioral symptoms. Nabilone 0.5 mg was introduced once daily and later increased to bid administration. Clinicians observed dramatic improvement of agitation and restlessness within weeks and noted no emergent side effects during three months continuous treatment.

All reports stated improvements of behavioral disturbances after oral administration of nabilone or dronabinol. It remains unclear, how the behavioral changes in the late dementia stages are modulated by CB_1_/CB_2_ agonists. Data from various animal models suggest that feeding behavior, sleep induction, circadian rhythm and serotonergic transmission are modulated via CB_1_ receptor agonism [115,116,117,118,119]. We found no report on CBD in AD and neither did we find a current clinical trial in the registries.

#### 3.2.2. Vascular dementia

Currently, there are no studies or case reports on cannabinoids in patients with vascular dementia. However, one of the six participants of the study by Walther *et al*. [112] was suffering from vascular dementia and improved during dronabinol treatment. The scarcity of reports on cannabinoid use in these patients may be a result of the symptoms presented. Patients with vascular dementia frequently suffer from apathy (65%), depression (45%), irritability (42%), and agitation (40%) [120]. Still, the literature suggests positive effects of cannabinoids in the pharmacotherapy of depression [121]. 

#### 3.2.3. Huntington’s disease

We could identify two clinical trials and two case reports of cannabinoid treatment in Huntington’s disease. Nabilone was the cannabinoid investigated in most reports. In fact, a randomized placebo controlled double blind crossover trial over five weeks each of nabilone 1 or 2 mg/d in 44 patients with HD found strong effects for nabilone on cognition, behavior and chorea symptoms [122]. In total, seven patients were withdrawn during the trial; some for adverse effects including suicidal ideation in one patient. However, in the other patients nabilone was well tolerated.

An early report of a randomized, placebo controlled, double blind crossover trial of CBD (10 mg/kg/d) for six weeks in 15 patients with HD failed to detect any effect [123]. CBD was neither toxic nor efficient in reducing symptoms of HD.

In a case report, a 42 year old woman with chorea Huntington history of 19 years and marked behavioral disturbances (agitation, impatience, rejection of care) acutely improved after smoking cannabis [124]. Later, the general practitioner administered nabilone 1 mg/d, which led to further improvements in behavior and chorea. 

Conversely, a 58 year old man with Chorea Huntington symptoms for six years, could not benefit from a single 1.5 mg nabilone administration [125]. Chorea symptoms as assessed before and after administration deteriorated for the following 24 hours.

The CB_1_/CB_2_ agonist nabilone reduced behavioral symptoms and choreatic movements in HD. However, in the case report of the 58 year old man, chorea worsened after a single administration. CBD instead had no effect.

#### 3.2.4. Parkinson’s disease

A survey in PD patients (age 45–83 years) suggested that 25% have used cannabis to treat symptoms [126]. In 45% of these cannabis users PD symptoms such as rigidity, tremor, bradykinesia and dyskinesia improved. Indeed, dyskinesia has been the primary target symptom of cannabinoid treatment approaches in PD. An open label study of CBD over six weeks in five patients with various etiologies of dyskinesias demonstrated improvement of dyskinesia between 20–50% [127]. The only PD patient improved 50% in terms of dyskinesia and worsened after cessation of CBD, however he experienced slight exacerbation of hypokinesia and tremor. Two double-blind, placebo-controlled, randomized crossover trials were performed to investigate the effect of cannabinoids on levodopa–induced dyskinesia. Oral nabilone (0.03 mg/KG) reduced dyskinesia by 22% in seven patients in a levodopa challenge test [128]. Nabilone was well tolerated and had no intrinsic antiparkinson action. In contrast, oral cannabis extract (2.5 mg Δ^9^-THC and 1.25 mg CBD) administered for four weeks in 19 PD patients although well-tolerated had no effect on dyskinesia [129]. The contradictory findings may be a result of the substances used (CB_1/2_ agonism *vs*. a combination of CB_1/2_ agonism and CBD), the administration period (once *vs*. four weeks) or a result of skewed data given the small sample size in the first trial [128]. Taken together, results are neither encouraging enough to support the use of cannabinoids in dyskinesia in PD [129], nor in primary dystonia [106]. 

In an exploratory randomized, double blind, placebo-controlled study, the CB_1_ antagonist SR 141716 failed to improve motor dysfunction or dyskinesia in PD after 16 days [130]. However, the number of patients on the active compound was very low (n = 4). 

Finally, a recent study investigated the effect of CBD on psychotic symptoms in PD [131]. The open label administration of oral flexible dose CBD (mean 400 mg/d) in six PD patients who had experienced psychotic symptoms for more than three months led to a significant decrease in psychopathological scales with most effect on delusions, thought disorder and retardation. Thus, CBD has some potential to become an alternative to antipsychotic drugs for psychosis in PD.

## 4. Conclusions

Several lines of evidence have demonstrated the role of cannabinoids in dementia. Cannabinoids seem to be involved in disease pathology in various ways, and some compounds were suggested to have therapeutic potential in neurodegenerative diseases. For instance, CB_1_/CB_2_ agonists may interrupt excitotoxicity and reduce neuroinflammation in AD brains, modulators of endocannabinoid signaling may reduce hyperactivity in HD, while CB_1_ agonists could reduce dyskinesia in PD. However, most of the *in vitro* findings need replication in animal studies and afterwards human trials are required.

In the field of human trials, curative or disease modifying approaches have not been followed yet. An interesting study objective would be to investigate in a prospective trial whether the non-psychoactive compound CBD may slow down the cognitive decline in AD. Furthermore, it should be evaluated whether the administration of CBD in combination with CB_1_ agonists or alone could slow the neurodegenerative process in patients suffering from HD and PD. Cannabinoid based drugs may therefore become a therapeutic option to modify the course of neurodegenerative diseases. 

The small but successful human trials with CB_1_ agonists in HD and AD that ameliorated behavioral disturbances are promising. The reported beneficial effects of Nabilone in HD or dronabinol in AD with behavioral disturbances call for replication in larger trials covering longer periods of observation. Given, that both substances prove to be save in long term administration, Dronabinol and Nabilone could soon become an adjunct treatment option in these severe conditions, *i.e.,* late stages of AD or HD with poor prognosis and behavioral disturbances. 

The transition of findings from bench to bedside and the extension of results from small clinical trials should be on the research agenda for the near future. Because treatment strategies for dementia are so preliminary at the current state of knowledge and the need for a cure is so desperate, it is worth pursuing the quest for one or more cannabinoid compounds in the field.
